# Genomic Surveillance of Vancomycin-Resistant Enterococcus faecium Reveals Spread of a Linear Plasmid Conferring a Nutrient Utilization Advantage

**DOI:** 10.1128/mbio.03771-21

**Published:** 2022-03-28

**Authors:** Mathilde Boumasmoud, Vanina Dengler Haunreiter, Tiziano A. Schweizer, Lilly Meyer, Bhavya Chakrakodi, Peter W. Schreiber, Kati Seidl, Denise Kühnert, Roger D. Kouyos, Annelies S. Zinkernagel

**Affiliations:** a Department of Infectious Diseases and Hospital Epidemiology, University Hospital Zurich, University of Zurich, Zurich, Switzerland; University Medical Center Utrecht; Emory University

**Keywords:** *Enterococcus faecium*, transmission networks, linear plasmid, N-acetyl-galactosamine, horizontal gene transfer

## Abstract

Healthcare-associated outbreaks of vancomycin-resistant Enterococcus faecium (VREfm) are a worldwide problem with increasing prevalence. The genomic plasticity of this hospital-adapted pathogen contributes to its efficient spread despite infection control measures. Here, we aimed to identify the genomic and phenotypic determinants of health care-associated transmission of VREfm. We assessed the VREfm transmission networks at the tertiary-care University Hospital of Zurich (USZ) between October 2014 and February 2018 and investigated microevolutionary dynamics of this pathogen. We performed whole-genome sequencing for the 69 VREfm isolates collected during this time frame and assessed the population structure and variability of the vancomycin resistance transposon. Phylogenomic analysis allowed us to reconstruct transmission networks and to unveil external or wider transmission networks undetectable by routine surveillance. Notably, it unveiled a *persistent clone*, sampled 31 times over a 29-month period. Exploring the evolutionary dynamics of this clone and characterizing the phenotypic consequences revealed the spread of a variant with decreased daptomycin susceptibility and the acquired ability to utilize N-acetyl-galactosamine (GalNAc), one of the primary constituents of the human gut mucins. This nutrient utilization advantage was conferred by a novel plasmid, termed pELF_USZ, which exhibited a linear topology. This plasmid, which was harbored by two distinct clones, was transferable by conjugation. Overall, this work highlights the potential of combining epidemiological, functional genomic, and evolutionary perspectives to unveil adaptation strategies of VREfm.

## INTRODUCTION

Enterococcus faecium is a gut commensal that has globally emerged as a leading cause of health care-associated infections in the past 20 years (1). E. faecium has substantially diversified into ecotypes adapted to environmental niches, one of which is the hospital (2, 3). Worldwide, strains causing hospital-acquired infections, such as endocarditis or urinary infections, most often belong to a single spreading lineage, designated clade A1 (2). Strains belonging to this lineage have accumulated multiple resistance genes, including the *van* gene operons, which confer resistance to vancomycin (1, 4).

Prevalence of vancomycin resistance is increasing worldwide, making vancomycin-resistant *E. feacium* (VREfm) a top priority pathogen for the development of new treatment options by the World Health Organization (5). In Switzerland, the first VREfm hospital outbreaks were reported in 2010 (6, 7) and followed by a marked increase in cases (8–11). Successive waves with specific clones propagated regionally, as in other countries (12, 13).

Recent genomic approaches have contributed to a better understanding of the mechanisms underlying VREfm dissemination. Expansion of vancomycin resistance is driven by both *de novo* generation and clonal spread of resistant strains (14, 15). Hospitals host multiclonal VREfm populations, introduced by multiple events (16). VREfm asymptomatic colonization, rarely identified in routine settings, is epidemiologically significant, as it represents a reservoir for transmission (17, 18) and often precedes infection (19, 20). During asymptomatic colonization, which can persist for several months and involve multiple strains (18, 19), E. faecium can evolve dynamically, with frequent gain and loss of genes (21). This phase is likely critical for adaptation of the pathogen because innovative traits introduced by horizontal gene transfer allow clones to adapt to environmental challenges and capitalize on the available growth resources (22).

Adaptation of E. faecium has been studied from a macroevolutionary perspective, and a central role for carbohydrate utilization genes has been identified in the speciation of the *Enterococcus* genus (23) as well as further differentiation of E. faecium lineages (2, 24–27). In particular, phosphotransferase systems (PTS), which couple the uptake and phosphorylation of carbohydrates (28), seem to play a pivotal role in adaptation. Notably, one PTS highly enriched in clinical isolates (PTS^clin^) is involved in intestinal colonization (27). However, at a microevolutionary scale, the gene flow experienced by individual clones in the hospital environment has been less extensively studied, and the functional consequences of the detected changes remain largely unknown (21). While a crucial role for E. faecium niche adaptation has been attributed to plasmids, most plasmid genes are poorly characterized (24).

In this study, we retrospectively characterized VREfm transmission networks at the tertiary-care University Hospital of Zurich (USZ) between 2014 and 2018 using a genomic approach. We explored evolutionary dynamics of a persistent VREfm clone. Notably, we identified and characterized a novel linear plasmid carrying carbohydrate utilization genes.

## RESULTS

### A multiclonal population dominated by one clone.

**FIG 1:**
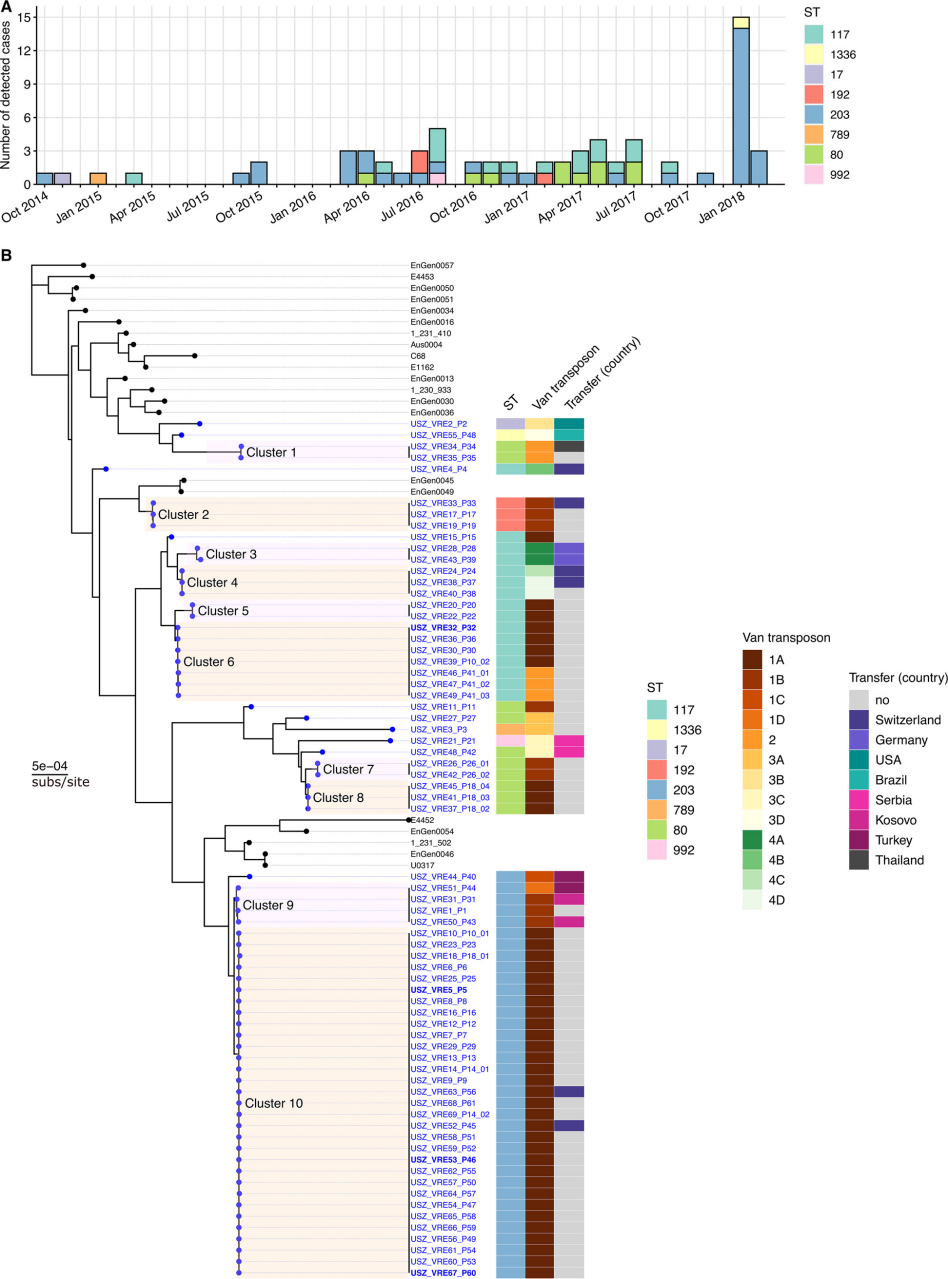
Epidemiology and population structure of VREfm at the University Hospital of Zurich (USZ). (A) All VREfm cases detected at the USZ between October 2014 and March 2018. The colors represent the multilocus sequence type (ST) of each isolate. (B) Maximum-likelihood phylogenetic tree based on the core gene alignment (1,546,174 bp), rooted using the clade regrouping EnGen0051, EnGen0050, and EnGen0057, with E4453 as an outgroup. The tips of the phylogeny marked with black dots correspond to 21 isolates collected worldwide (2) (Data Set S1, Tab 2). Those marked with blue dots correspond to the 69 isolates of this study. The labels of the latter indicate the isolation location, USZ, the unique isolate identifier (ID), VRExx, and the ID of the patient from whom it was isolated, Pxx. In the few cases in which multiple isolates were derived from the same patient, the last number is the index of each isolate within the longitudinal series. The four isolates with boldface labels were additionally subjected to long-read sequencing to complete their assembly. Clusters, shaded with alternating colors for a clear visualization, were defined based on distances in this core gene alignment (Data Set S1, Tabs 3 and 4). The first column of the color map shows the ST of each isolate. The second column shows the variant of the vancomycin transposon. Three different types of transposon Tn*1546*, carrying the *vanA* genes, are labeled 1 to 3, and the subtypes they comprise further distinguished with letters and each highlighted with a different shade ranging from dark brown to light yellow. The transposon Tn*1549* carrying the *vanB* genes is labeled 4 and the four subtypes it contains further distinguished with letters and highlighted with green shades. The third column of the color map shows which patients were admitted at USZ upon transfer from another hospital and the corresponding country of the hospital.

Between 2011 and 2014, ≤3 cases of infection or colonization by VREfm were detected each year at the USZ. Since 2014, the detected VREfm cases have increased steadily (Fig. 1A). Between October 2014 and February 2018, a total of 69 VREfm isolates were collected from 61 patients. They were derived from both colonizing (*n* = 45) and infecting strains (*n* = 24) and had been sampled from different materials and body sites, such as urine, feces, abdominal cavity, surgical sites, wounds, and occasionally bloodstream (see Data Set S1 in the supplemental material, Tab 1). Based on a combination of epidemiological criteria and traditional genotyping (multilocus sequence typing [MLST] and pulsed-field-gel electrophoresis [PFGE]), some transmission events were suspected, prompting the screening of exposed patients. Notably, in January and February 2018, six patients suffered from various infections and 11 patients were found to be colonized with VREfm (Fig. 1A). Traditional genotyping indicated clonality of the 17 isolates, and an epidemiological link was established across all but one of the patients.

10.1128/mbio.03771-21.1 DATA SET S1 Additional tables containing metadata for the collection of isolates of this study and reference genomes (Tabs 1 and 2), phylogenomic clusters details (Tabs 3 and 4), list of mutations differentiating the *persistent clone* isolates (Tab 5), annotation of pELF_USZ CDS (Tab 6), comparison with other plasmid sequences (Tab 7), homologous gene clusters query (Tab 8), and sequence quality metrics (Tab 9). Download Data Set S1, XLSX file, 0.08 MB. Copyright © 2022 Boumasmoud et al. 2022 Boumasmoud et al. https://creativecommons.org/licenses/by/4.0/ This content is distributed under the terms of the Creative Commons Attribution 4.0 International license.

In this study, we retrospectively sequenced the whole genome of the 69 VREfm isolates. Comparison with the diverse collection of 73 E. faecium genomes from Lebreton et al. (2) (Data Set S1, Tab 2) demonstrated that all isolates clustered within clade A1 of human-infecting strains (https://microreact.org/project/VREfm_USZ_in_global_context).

Thus, we constructed a maximum core genome tree with the 21 clade A1 genomes (Fig. 1B, https://microreact.org/project/VREfm_at_USZ). This revealed the striking domination of a single clone within a multiclonal population. While most phylogenomic clusters contained two to seven isolates, *cluster 10* contained 31 isolates: the 17 isolates of the 2018 outbreak as well as 14 isolates from 2015 and 2016 (Data Set S1, Tab 1).

To explore routes of vancomycin resistance spread, we assessed diversity of the vancomycin resistance transposon within the collection. Based on insertion sequences incorporated and similarity to the reference *vanA* and *vanB* transposons, 13 distinct transposon variants were identified (Fig. S1). Within clusters, the isolates’ transposon variants were highly concordant, illustrating clonal spread of vancomycin resistance (Fig. 1B). Across clusters, some transposon variants were shared, suggesting that vancomycin resistance spread horizontally as well.

10.1128/mbio.03771-21.3 FIG S1 Vancomycin transposon variability. Alignment to Tn1546 of B4147 (76, 77) for isolates with *vanA* genes (A) and to Tn*1549* of Aus0004 (14) for isolates with *vanB* genes (B). Within each panel, isolates are ordered based on their position in the phylogenetic tree of Fig. 1B. The *x* axis shows the position on the reference in bp. Based on genotype (*vanA* or *vanB*) and insertion sequences incorporated, four transposon types (labeled 1 to 4) were defined, and based on similarity with the references, visualized here for each isolate, these were subdivided into subtypes (labeled A to D). The horizontal segments indicate covered areas, vertical black lines represent SNPs, and the triangles represent insertion sequences. Distinct variants of the vancomycin transposon are each attributed a color (brown shades for *vanA*, type 1 to 3, and green shades for *vanB*, type 4), taken up in Fig. 1B. All *vanA* transposon types exhibited full or partial loss of *orf1* and *orf2* due to the incorporation of insertion sequences. In contrast, all *vanB* transposon types covered the 33,810-bp Tn*1549* reference except for the loss of the *vanR* and *vanS* genes by one isolate (USZ_VRE24_P24). Download FIG S1, JPG file, 2.2 MB. Copyright © 2022 Boumasmoud et al. 2022 Boumasmoud et al. https://creativecommons.org/licenses/by/4.0/ This content is distributed under the terms of the Creative Commons Attribution 4.0 International license.

### Transmission networks revealed by phylogenomic analysis.

Next, we performed full-genome comparisons of the isolates within each cluster and filtered single-nucleotide polymorphisms (SNPs) resulting from recent recombination events. Isolates suspected to be linked based on the routine surveillance approach (combining epidemiological criteria and traditional genotyping) differed pairwise by as much as 8 SNPs. In addition, several isolates not suspected to be linked based on routine surveillance were revealed to be closely related (0 to 63 SNPs; Data Set S1, Tab 3 and 4, Fig. S2), likely reflecting indirect epidemiological linkage involving longer transmission chains.

10.1128/mbio.03771-21.4 FIG S2 Colormap of within-cluster pairwise SNP distances based on the recombination-filtered alignment. A log_10_ transformation is performed on the color scale to allow the observation of differences across lower values. All pairwise distances >12 SNPs are annotated in the corresponding tile. Pairs of isolates suspected to be linked based on the routine surveillance approach are labelled with a white cross. All these cases displayed <8 SNPs pairwise distance (Data Set S1, Tab 4). Download FIG S2, JPG file, 1.8 MB. Copyright © 2022 Boumasmoud et al. 2022 Boumasmoud et al. https://creativecommons.org/licenses/by/4.0/ This content is distributed under the terms of the Creative Commons Attribution 4.0 International license.

To identify potential introduction of VREfm from external settings, we assessed which patients had been admitted to USZ after having been hospitalized in another hospital (Fig. 1B). For all transfer patients, VREfm was first detected at the USZ, sometimes multiple weeks after admission and/or after a first negative screening, except for patient 39, for whom VREfm colonization was known prior to transfer to our hospital (VRE43_P39, *cluster 3*). Thus, in most cases VREfm acquisition is likely to have happened internally. However, on three occasions, we observed an association between the country of previous hospitalization and either the isolates’ core genomes (*cluster 3* and *cluster 9*) or their vancomycin resistance transposon (VRE21_P21 and VRE48_P42), supporting the hypothesis of repeated sampling from external transmission networks. For *cluster 3*, containing two isolates sampled from patients previously hospitalized in Germany (VRE28_P28 and VRE43_P43), this hypothesis was further supported by their close relatedness to an isolate sampled in 2017 at a tertiary care hospital of the Rhine-Main metropolitan area (29) (VRE01-01; 18 and 38 SNPs, respectively).

In conclusion, phylogenomic analysis allowed us to unveil indirect epidemiological linkage undetectable by routine surveillance.

### Vertical evolution during longitudinal surveillance.

**FIG 2:**
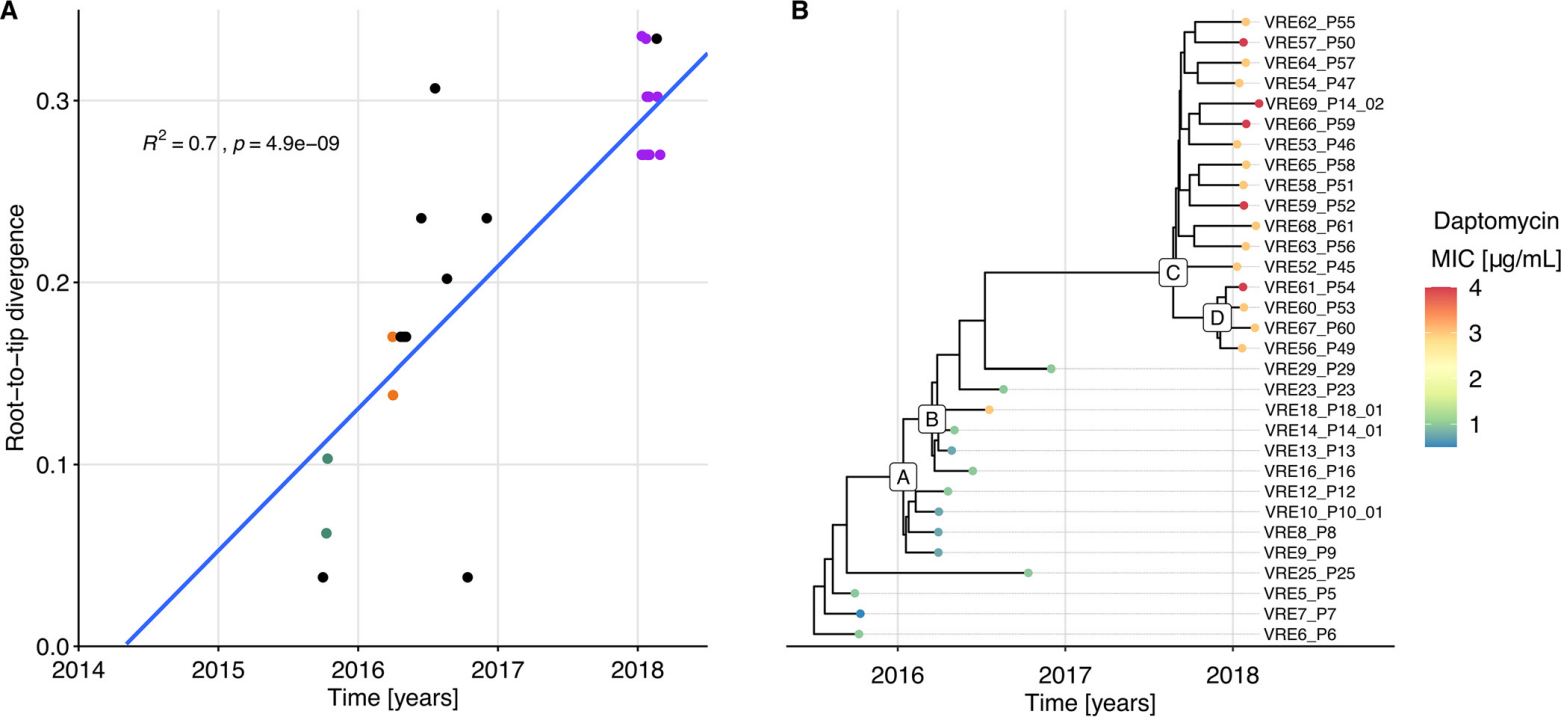
Vertical evolution of the *persistent clone*. (A) Regression of the root-to-tip divergence against sampling date, based on the phylogeny resulting from the recombination-free SNP alignment of the isolates from the *persistent clone*. *R*^2^ and *P* values are based on a Pearson correlation. Divergence is based on the 32-bp SNP alignment, so 0.32 corresponds to 10 SNPs. Dots corresponding to isolates from patients with an established epidemiological link share the same color, e.g., all but one of the 2018 outbreak’s isolates are in purple. All isolates from patients without established epidemiological link are in black. (B) Time-scaled maximum clade credibility (MCC) tree inferred by Bayesian analysis. The color of the dots marking the tips of the phylogeny reflects the degree of susceptibility to daptomycin (MIC). Letters labeling selected internal nodes are meant as a reference to specific clades, referred to in Data Set S1, Tab 5.

We subsequently focused on *cluster 10*, representing the dominant clone repeatedly sampled (*n* = 31, between September 2015 and February 2018), further referred to as the *persistent clone*. Transmission events supported by epidemiological links and consistent typing information (MLST and PFGE) had been established in three instances along this transmission chain. Overall, the sampled isolates differed pairwise by up to 12 SNPs in their core genome. The temporal signal of the phylogeny resulting from the recombination free alignment was strong, as indicated by the correlation between root-to-tip divergence and sampling time (Fig. 2A). Therefore, we performed Bayesian phylogenetic analysis to estimate the substitution rate of this clone, obtaining a rate of 3.8 substitutions per genome per year (95% credibility interval, 2.3, 5.5).

Mapping each isolate to the fully assembled VRE5_P5 genome allowed us to establish a catalogue of all mutations differentiating the 31 isolates (Data Set S1, Tab 5). The clade grouping the isolates of the outbreak of 2018 (*outbreak clade*, originating from node C in Fig. 2B) was characterized by six mutations compared to the *ancestral* isolates (Table 1).

**TABLE 1:** Mutations characteristic of the *outbreak clade* originating from the node labeled C in Fig. 2B^*a*^

Position in VRE_5_P5 (bp)	Position in Aus0004 (bp)	Type	Length (bp)	Effect	Nucleotide change	Amino acid change	Gene	Protein
60123	95524	SNP	1	Missense variant	2032G>A	Val678Ile	*fusA*	Elongation factor G
389298	416003	ins	12	Conservative inframe insertion	536_537insCGAAGACAGCAA	Asn179_Glu180, ins, GluAspSerAsn	*malP*	Glycoside hydrolase, family 65
1666026	1271778	del	1	Frameshift variant	1187delA	Lys399fs	*mvaE*	Hydroxymethylglutaryl-coenzyme A reductase
1672764	1278516	SNP	1	Missense variant	59C>A	Ala20Asp	*cls*	Cardiolipin synthase
2013711	2022517	SNP	1	Missense variant	1261G>A	Ala421Thr	*brnQ*	Branched-chain amino acid transport system II carrier protein
pl1, 184545	—	del	63	—	—	—		—

a The mutation with no effect (—) was in an intergenic region on plasmid 1 (pl1). All other mutations were chromosomal and nonsynonymous. Del, deletion; ins, insertion.

One of these mutations was in the *cls* gene, which encodes cardiolipin synthase (Cls), an enzyme involved in phospholipid metabolism. Mutations in this gene have been associated with decreased susceptibility to daptomycin, one of the last-resort drugs used to treat VREfm (30, 31). The Cls substitution Ala20Asp, characterizing the *outbreak clade* isolates, was previously reported in an E. faecium clinical isolate belonging to an isogenic pair differing in daptomycin susceptibility (32). In accordance with this, the daptomycin MIC was 3 to 4 μg/mL for the *outbreak clade* isolates and 0.5 to 1 μg/mL for the *ancestral* isolates, except for VRE18_P18, for which the daptomycin MIC was 3 μg/mL (Fig. 2B and Data Set S1, Tab 1). This variant was probably selected within-host during daptomycin treatment. Among the sampled patients, P23 was treated after sampling in 2016 and P45, P52, and P65 were treated prior to sampling in 2018 (Fig. S3).

10.1128/mbio.03771-21.5 FIG S3 Daptomycin MIC. E. faecium strains with daptomycin MIC falling in the range ≤4 μg/mL (color highlighted area) are considered susceptible-dose-dependent while those with MIC ≥8 μg/mL (shown with a dashed line) are considered nonsusceptible according to the CLSI guidelines of 2019 (99). The same data are presented in panels A and B. In both panels, color of data points reflects the clusters. Panel A highlights changes in the MIC within clusters. To this scope, isolates are presented in the order in which they appear in the phylogenetic tree of Fig. 1B. Shape of the data points illustrates whether the patient from whom the isolate was sampled had been treated with daptomycin after, before, before and after or not at all. The two cases including “before” are highlighted with a closed shape, indicating that the isolate was derived from a strain which had experienced antibiotic pressure within host. Panel B shows the global temporal evolution. Download FIG S3, JPG file, 1.0 MB. Copyright © 2022 Boumasmoud et al. 2022 Boumasmoud et al. https://creativecommons.org/licenses/by/4.0/ This content is distributed under the terms of the Creative Commons Attribution 4.0 International license.

An additional *outbreak clade*-specific mutation was an SNP in *fusA*, the gene encoding elongation factor G (EF-G). Mutations in *fusA* are associated with resistance to fusidic acid (33–35). However, this specific mutation did not affect the fusidic acid MIC, which was 0.19 μg/mL for both VRE5_P5, *ancestral* isolate, and VRE53_P46, *outbreak clade* isolate.

In conclusion, tracking the vertical evolution of the *persistent clone* based on genomes of isolates sampled from different patients at different time points revealed that only a few mutations accumulated over time, including an SNP leading to decreased daptomycin susceptibility.

### Horizontal evolution: acquisition of a novel plasmid.

**FIG 3:**
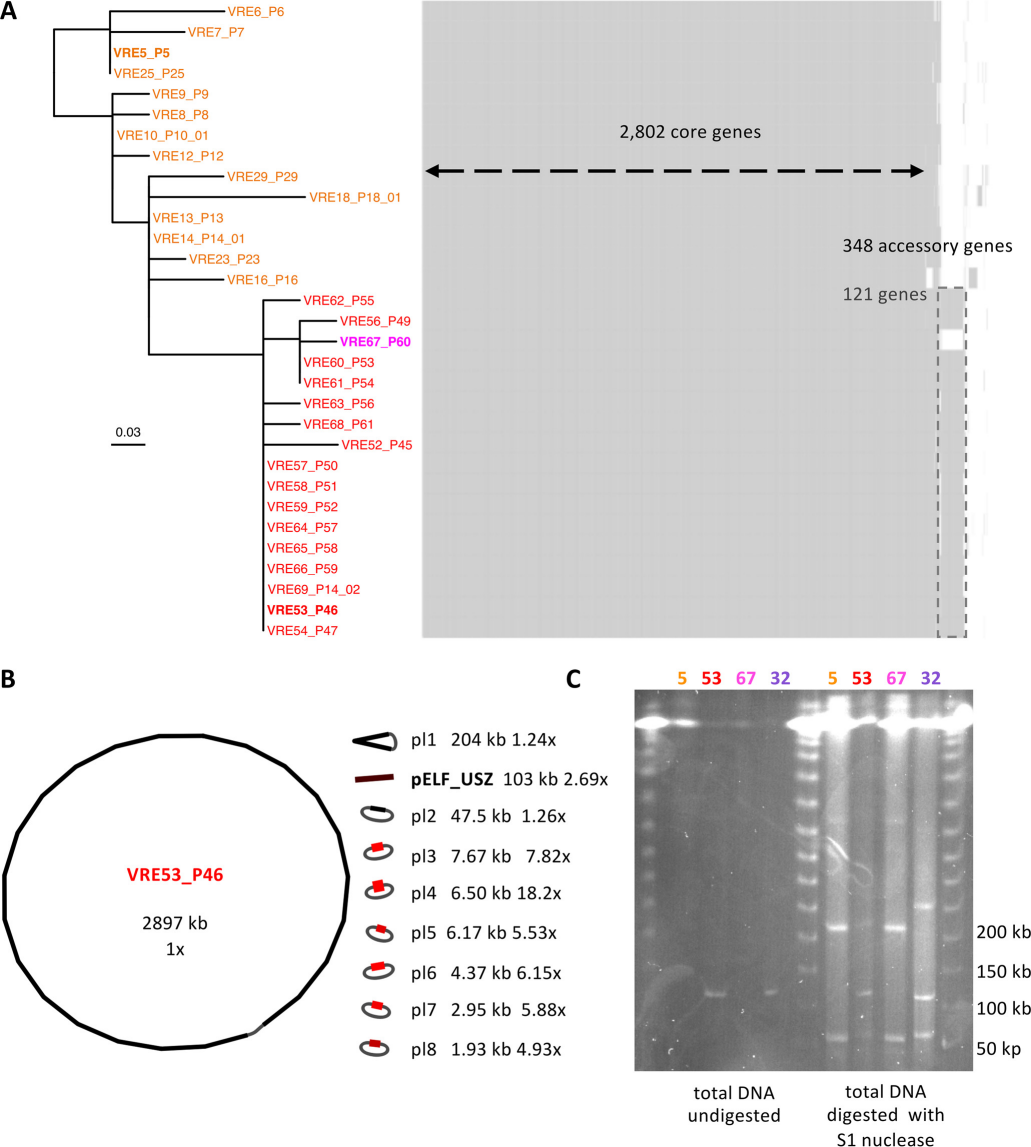
Content and structure of the genome of isolates representing the *persistent clone*. (A) Maximum likelihood phylogenetic tree along with gene content map. The phylogeny is based on the recombination-free SNP alignment (32 bp). The *ancestral* isolates are labeled in orange, the *outbreak clade* isolates are labeled in red, except for the only clade member that did not carry the additional mobile genetic element, labeled in magenta. The three isolates chosen as representatives for further experiments are highlighted in boldface. Each isolate has its corresponding row in the gene content map, where each column represents one gene. (B) Visualization of the complete assembly of VRE53_P46. The length of each sequence is given in kilobase pairs (kb). Next, the coverage (depth) with respect to the chromosome coverage (set to 1×) was determined. The color, ranging from black, for the chromosome, to light red reflects the coverage and a gray string indicates circularization. (C) PFGE of three representative isolates of the *persistent clone*: VRE5_P5, VRE53_P46 and VRE67_P60 (labeled 5, 53, and 67, respectively), as well as one isolate from cluster 6, VRE32_P32 (labeled 32). Four columns on the left, undigested total DNA; four columns on the right, total DNA digested by S1 nuclease, for plasmid linearization.

Next, we focused on changes in genome content, since the acquisition of accessory genes has the potential to confer new traits to a bacterial strain, which may be advantageous in given environments. We observed both the acquisition and the loss of genes. The most notable difference among the *persistent clone* isolates, which shared 2,802 genes, were 121 genes specific to the *outbreak clade* isolates, except for VRE67_P60 (Fig. 3A).

Most of these accessory genes had their product annotated as *hypothetical protein.* Among the 22 genes with a putative product, there was a cluster of 11 consecutive genes encoding proteins involved in carbohydrate uptake and metabolism and transcriptional regulators (Data Set S1, Tab 6). The complete set of accessory genes was also present in two isolates from another genetic background (VRE32_P32 and VRE46_P41_01, *cluster 6*, ST-117; Fig. S4). In the short-read assemblies, this set of genes was distributed on three to four closely connected contiguous sequences (*contigs*) and exhibiting a coverage 2- to 3-fold higher than the assembly median coverage (Fig. S5A). This suggested that they represented a plasmid sequence.

10.1128/mbio.03771-21.6 FIG S4 Map of accessory genes. The maximum-likelihood phylogenetic tree of Fig. 1B is shown on the left and a map of accessory genes, i.e., genes present in >14% and <99% genomes of the collection is shown on the right. The pattern of presence/absence is shown with vertical segments (black/white, respectively) for each row, which corresponds to one isolate (tree tip). Arrows at the bottom of this map point towards specific gene clusters. These represent two carbohydrate utilization operons: the one including PTS^clin^, the mannose family PTS previously shown to be enriched in clinical isolates (highlighted in green) (27), and the cargo genes of pELF_USZ, which included a mannose family PTS as well (highlighted in cyan). Download FIG S4, JPG file, 1.8 MB. Copyright © 2022 Boumasmoud et al. 2022 Boumasmoud et al. https://creativecommons.org/licenses/by/4.0/ This content is distributed under the terms of the Creative Commons Attribution 4.0 International license.

10.1128/mbio.03771-21.7 FIG S5 Plasmids in fragmented and complete assemblies. (A) Short-read assembly graphs of USZ_VRE53_P46 and USZ_VRE32_P32. For each isolate, the same assembly graph is shown twice: on the left, color reflects the coverage (depth), ranging from black for median coverage, to light red for highest coverage; on the right, rainbow-colored contigs are BLAST hits of pELF_USZ to visualize its localization within the graph. (B) Matrix displaying the percent average nucleotide identity (ANI), followed by mash distance for each pair of plasmid sequences. Comparisons for which the ANI was of 0% are left blank. Four matching pairs (ANI > 99% and mash distance < 0.01) are highlighted in green. (C and D) Alignments of two pairs of matching plasmid sequences: pELF_USZ and pl2. A minimum blast length of 500 bp and minimum identity of 99% were considered to plot the alignment blocks. Coding sequences are shown with arrows and their color reflects their function. Cargo genes of pELF_USZ are highlighted in dark blue. Download FIG S5, JPG file, 1.7 MB. Copyright © 2022 Boumasmoud et al. 2022 Boumasmoud et al. https://creativecommons.org/licenses/by/4.0/ This content is distributed under the terms of the Creative Commons Attribution 4.0 International license.

To confirm this, we subjected four selected isolates to long-read sequencing. When combining short and long reads, the mobile genetic element detected in the short-read assemblies of VRE32_P32 and VRE53_P46 were assembled into uncircularized sequences of 102 and 103 kb, respectively (Fig. 3B and Fig. S5B). Since Unicycler (36), the assembler we employed, attempts to circularize each contig, we assumed that these uncircularized sequences were either the result of sequencing/assembly artifacts or of a biological linear topology. We considered the first interpretation less plausible, as the phenomenon was consistent across two different strains/assemblies. Accordingly, we carried out molecular analyses to evaluate the possibility of a linear topology.

PFGE of the total DNA digested with S1 nuclease, which linearizes supercoiled plasmids by unspecific cleavage, revealed bands at the expected size based on the assembled plasmid sequences. One of these was at around 100 kb, corresponding to the size of the uncircularized plasmid sequence (Fig. 3C). In parallel, the control consisting of undigested total DNA also showed the same band for the isolates VRE32_P32 and VRE53_P46, supporting an original linear form. Taking up the nomenclature *pELF*, for plasmid element linear form, introduced for the first reported E. faecium linear plasmids (37, 38), we termed the linear plasmids detected in this study pELF_USZ.

### Sharing of plasmids between clones.

Next, we evaluated the similarity between the two linear plasmid sequences as well as all other assembled plasmid sequences of the two isolates from distinct clones (VRE32_P32 and VRE53_P46). This revealed that the two linear plasmids, denoted pELF_USZ in both assemblies, the vancomycin resistance plasmids, denoted pl2 in both assemblies, and two smaller plasmids, denoted pl6, pl8 in VRE53_P46 and pl4, pl7 in VRE32_P32, were identical or identical except for small insertions or inversions (Fig. S5B).

Notably, there was a difference in length of 1.5 kb and 3.8 kb between the two linear plasmids and the two vancomycin resistance plasmids, respectively. In the first case, this was due to the incorporation of an insertion sequence (IS256) between two coding sequences of VRE53_P46 pELF_USZ (Fig. S5C). In the second, it was due to the addition of a *pseudo compound transposon*, three coding sequences (CDSs) flanked by two directly oriented identical copies of the insertion sequence IS1216 in VRE32_P32 pl2 in place of a single copy of the same IS1216 in VRE53_P46 pl2 (Fig. S5D). The conversion between these two configurations is known to occur with a single conservative transposition event (39, 40).

The fact that two isolates from distinct clones shared four plasmids suggested they had horizontal gene transfer opportunities despite having been sampled 1 year apart from patients without an epidemiologic link.

### Genetic structure of the novel linear plasmid pELF_USZ.

**FIG 4:**
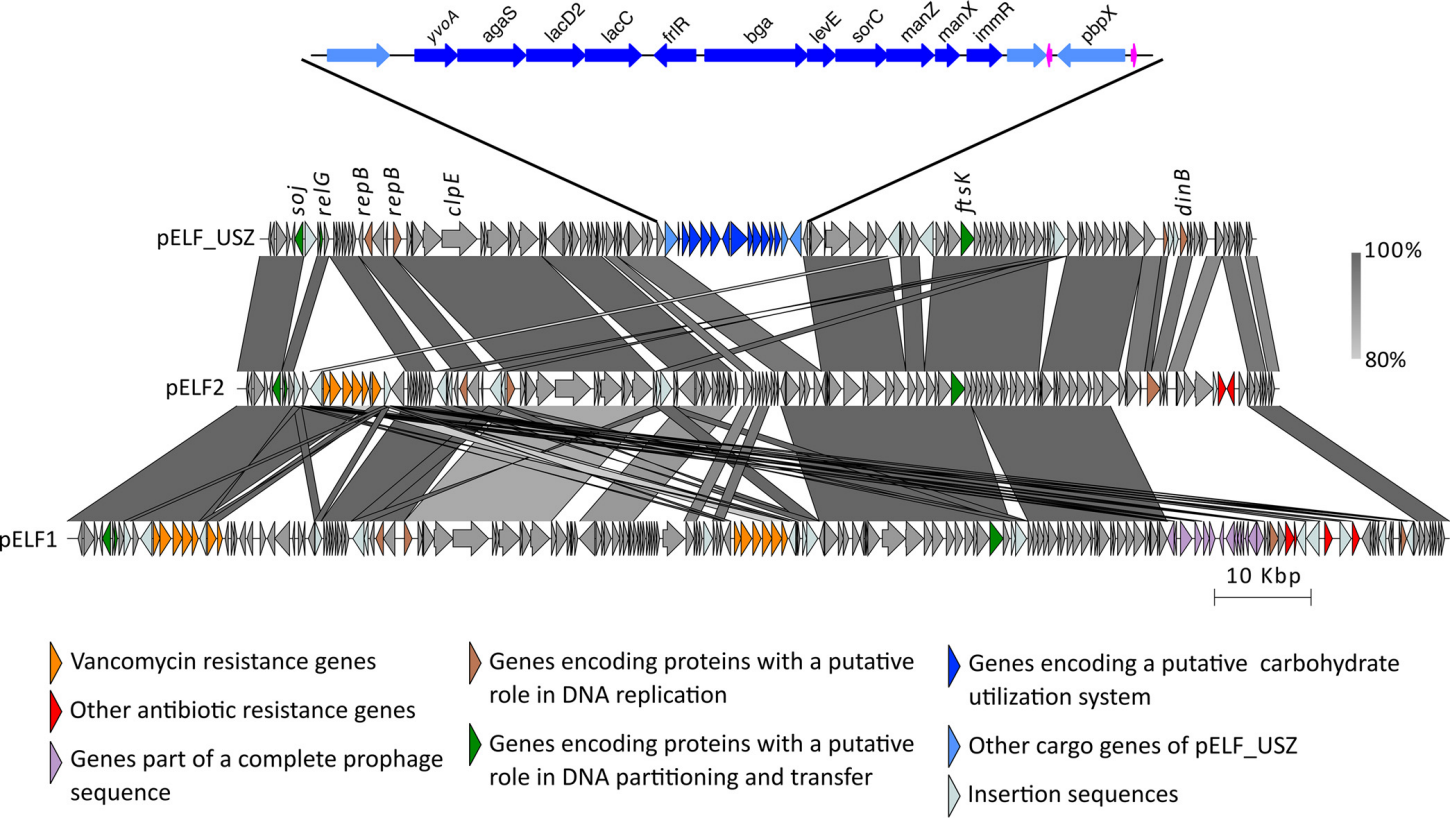
Genetic structure of pELF_USZ. Adapted and expanded from Fig. 3 of Hashimoto et al. (37). Visualization of the multiple alignment of the three plasmid sequences. Regions shared between sequences are connected by vertical blocks. A minimum blast length of 500 bp and minimum identity of 80% was considered. Color gradient reflects percent identity. Coding sequences are shown with arrows, and their color reflects the genes’ functions. An intact phage identified on pELF1 is highlighted in purple, and the cargo genes of pELF_USZ are highlighted in blue. This 14-kb stretch is amplified for a clearer visualization. The 11 consecutive genes encoding a carbohydrate utilization system are in dark blue, and two tRNA coding genes are in magenta.

Comparing pELF_USZ with the enterococcal linear plasmids pELF1 and pELF2 (143,416 kb and 108,102 kb, respectively) revealed a clear homology (Fig. 4). The RAST annotation pipeline (41) identified on each of them two CDSs encoding putative replication initiation proteins, RepB, which belonged to the Rep_3 family. None of the CDSs displayed similarity to known relaxases, the other hallmark traditionally used for plasmid classification (42). Nevertheless, each plasmid encoded three proteins with a putative role in DNA partitioning and transfer: the sporulation initiation inhibitor protein Soj (ParAB), the translocase FtsK (SpoIII), and the toxin RelG (RelE/StBE) (Fig. 4 and Data Set S1, Tab 6).

The left extremity, reported to form a hairpin loop in pELF1 and pELF2, behaved similarly in pELF_USZ. Indeed, when assembling long reads only, the plasmid sequence was 143 kb, that is, 40 kb longer than that obtained upon hybrid assembly. Inspection of these extra 40 kb revealed that they corresponded to the inverse complement of the first 40 kb of pELF_USZ. Furthermore, the motif 5′-TATA-3′ was at the center of the loop resulting from the folding of these inverted repeats, as identified in pELF1 and pELF2 (Fig. S6A). When querying pELF_USZ against the nucleotide collection database (nr/nt), several E. faecium plasmids were identified as hits, and further inspection revealed that they corresponded to uncircularized sequences within their respective complete assemblies (Fig. S6B and Data Set S1, Tab 7).

10.1128/mbio.03771-21.8 FIG S6 Topology of pELF_USZ and homology with other plasmid sequences. (A) Alignment of the plasmid sequence resulting from a hybrid assembly with the corresponding segment in the long-reads-only assembly. Coding sequences are shown with arrows. A minimum blast length of 500 bp was considered to plot the alignment blocks. The arrow originating from the center of the inverse repeats indicates the DNA folding of the 113 bp spanning this area. (B) Multiple sequence alignment of the eight plasmid sequences corresponding to the best hits when querying pELF_USZ against the nucleotide collection database (nr/nt) as per 04.12.2020 (Data Set S1, Tab 7). In addition, pSRR24, despite not ranking in the top-8 hits, was the only E. faecium assembly including the cargo genes of pELF_USZ, highlighted in dark blue, as per 04.12.2020. Coding sequences are shown with arrows and their color reflects their function. Download FIG S6, JPG file, 2.1 MB. Copyright © 2022 Boumasmoud et al. 2022 Boumasmoud et al. https://creativecommons.org/licenses/by/4.0/ This content is distributed under the terms of the Creative Commons Attribution 4.0 International license.

Within this conserved backbone, the plasmids facultatively harbored a set of cargo genes. While pELF1 and pELF2 carried resistance determinants, including *van* gene operons, pELF_USZ carried a stretch of 14 kb consisting of two consecutive operons encoding a carbohydrate degradation pathway (*agaS*, *lacD2*, *lacC*, and *bga*) and a mannose-family PTS (*levE*, *sorC*, *manZ*, and *manX*) interleaved with transcriptional regulators of the GntR family (*yvoA* and *flrR*) and the Xre family (*immR*). These operons were framed by a transposase on one side and tRNA coding genes and *pbpX* on the other side (Fig. 4; Data Set S1, Tab 6). In contrast to the backbone, this set of cargo genes was not common in other E. faecium assemblies. A partial version, including the carbohydrate operons and transcriptional regulators (99.28% identity), was part of one E. faecium plasmid assembly, pSRR24 (43) (Fig. S6B).

In conclusion, the homology in sequence and topology of the backbone of pELF_USZ and other E. faecium plasmid sequences with the backbones of pELF1 and pELF2 suggested the existence of a conserved family of linear plasmids in E. faecium.

### Function and transferability of pELF_USZ.

**FIG 5:**
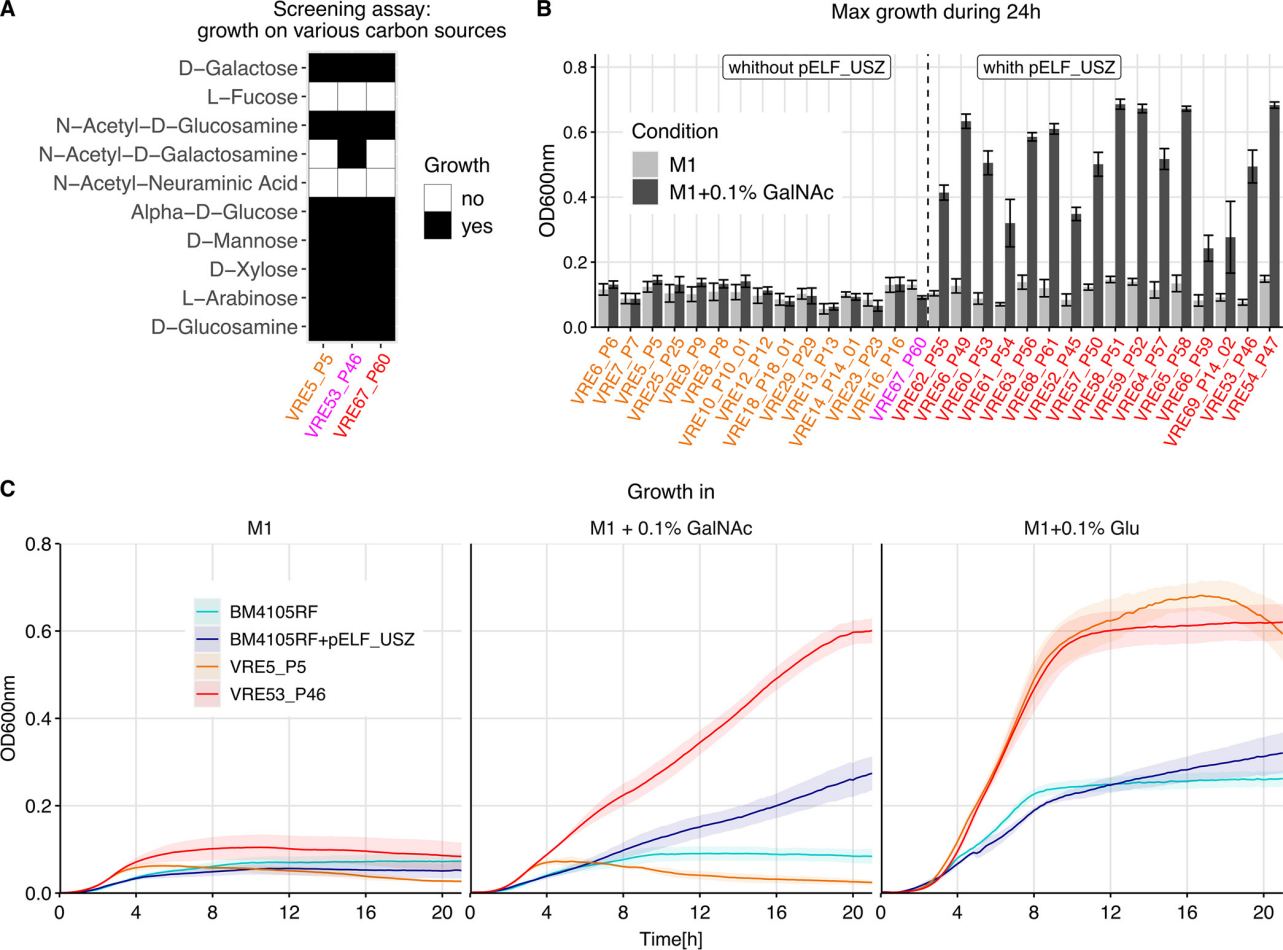
Function and transferability of pELF_USZ. (A) Growth in M1 supplemented with various carbon sources for three representative isolates of the *persistent clone*. This screening experiment was performed with the Phenotype MicroArrays PM1 and PM2A, Biolog Inc. Growth was measured by monitoring the optical density at 600 nm (OD_600_) during 48 h. Conditions yielding an area under the curve above an empirically determined threshold were considered growth permissive. Here, 10 out of the 190 conditions screened are displayed, selected based on their biologic relevance: the first five are monosaccharide components of mucins, and the following five are monosaccharide components of plant polysaccharides. (B) Maximum OD_600_ reached by the 31 isolates of the *persistent clone* during 24 h of growth in either M1 only or in M1 supplemented with 0.1% GalNAc (mean and standard error of mean are displayed, *N* ≥ 3). The color of the labels highlights the groups previously introduced: *ancestral* isolates are in orange and the *outbreak clade* isolates are in red, except for the only clade member that did not carry pELF_USZ, which is in magenta. The isolates are ordered based on their position on the phylogenetic tree of Fig. 3A, except for VRE67_P60, shifted to be grouped with the isolates that did not carry pELF_USZ. The two groups, comprising isolates with or without pELF_USZ, differed significantly in their growth in M1 supplemented with GalNAc, Welch’s *t* test *P* = 1.17e−08. (C) Growth curves in M1, M1 supplemented with 0.1% GalNAc, and M1 supplemented with 0.1% glucose (Glu) for the recipient strain BM4105RF, the transconjugant BM4105RF+pELF_USZ, and the two representative isolates of the *persistent clone*, VRE5_P5, which did not carry pELF_USZ, and VRE53_P46, which did carry pELF_USZ. The average from three biological replicates is shown, and the shaded area corresponds to the standard error of the mean.

To determine the role of the carbohydrate utilization genes of pELF_USZ, we screened three representative isolates of the *persistent clone* for their ability to utilize 190 different carbon sources. We used the semidefined medium M1, which minimizes growth of E. faecium when no carbon source is added (44). We found that 49 carbon sources sustained growth of all three isolates (Fig. S7). Furthermore, glycerol sustained the growth of the two *outbreak clade* isolates VRE53_P46 and VRE67_P60 but not of the *ancestral* isolate VRE5_P5. We attributed this difference and small differences in growth magnitude to the core genome mutations differentiating the three isolates. However, one condition distinguished the isolate harboring pELF_USZ (VRE53_P46) from those without pELF_USZ (VRE5_P5 and VRE67_P60): while the former could utilize *N*-acetyl-d-galactosamine (GalNAc) for growth in M1, the latter two could not (Fig. 5A). Further growth assays, including all isolates of the *persistent clone*, confirmed that none of the *ancestral* isolates could utilize GalNAc as the growth substrate, while all the *outbreak clade* isolates except for VRE67_P60 could, supporting that the growth advantage was conferred by pELF_USZ (Fig. 5B and Fig. S8A).

10.1128/mbio.03771-21.9 FIG S7 Utilization of 190 single carbon sources for representative isolates of the *persistent clone*. Conditions in which the area under the curve (AUC) was ≥230 were considered growth permissive. White crosses over a black square indicate false positives (AUC, within biological replicates standard error, <230). Download FIG S7, JPG file, 1.4 MB. Copyright © 2022 Boumasmoud et al. 2022 Boumasmoud et al. https://creativecommons.org/licenses/by/4.0/ This content is distributed under the terms of the Creative Commons Attribution 4.0 International license.

10.1128/mbio.03771-21.10 FIG S8 GalNAc utilization and growth in BHI. Growth in M1 medium and M1 medium supplemented with 0.1% GalNAc for the isolates of *cluster 10* (A) and *cluster 6* (B). In panel A, the *ancestral* isolates are in orange and the *outbreak clade* isolates are in red, except for the only clade member which did not carry pELF_USZ (VRE67_P60), which is in magenta. In panel B, the isolates which carried pELF_USZ are in purple (VRE32_P32 and VRE46_P41_01), while the others are in yellow (VRE30_P30, VRE36_P36, VRE39_P10_02, VRE47_P41_02, VRE49_P41_03). (C) Homologous gene clusters query in the VRE32_P32 genome. The second and third rows show two gene clusters homologous to the carbohydrate utilization system of pELF_USZ (first row). On the left, genomic location of each cluster, i.e, the scaffold (pELF_USZ, chr: chromosome, and pl1: plasmid 1) and position in bp on this scaffold. CDS are shown with arrows. CDS with hits in the identified homologous gene clusters are colored consistently across clusters, and their annotation is shown on the right. CDS shared between clusters are connected by vertical blocks with a grayscale that reflects % identity. (D) Maximal growth rate of the recipient and donor strains and the *persistent clone* isolates calculated based on the optical density measurements using 1-h intervals. Mean and standard error of mean of three biological replicates are shown. The isolates are ordered based on their position on the phylogenetic tree of Fig. 3A. The maximal growth rate of the two groups comprising *ancestral* isolates or *outbreak clade* isolates differed significantly (with mean ± standard error of mean 0.137 ± 0.003 h^−1^ and 0.165 ± 0.003 h^−1^, Welch’s *t* test *P* = 4.12 e−06). The maximal growth rate of the recipient (BM4105RF) and transconjugant (BM4105RF+pELF_USZ) did not significantly differ (0.208 ± 0.010 h^−1^ and 0.223 ± 0.008 h^−1^, average ± standard error of the three biological replicates). Download FIG S8, JPG file, 1.4 MB. Copyright © 2022 Boumasmoud et al. 2022 Boumasmoud et al. https://creativecommons.org/licenses/by/4.0/ This content is distributed under the terms of the Creative Commons Attribution 4.0 International license.

Outside *cluster 10*, only two other isolates of our collection carried pELF_USZ (VRE32_P32 and VRE46_P41_01). They belonged to *cluster 6*, which contained another five isolates without pELF_USZ, of which two were follow-up isolates from patient 41 (VRE47_P41_02 and VRE49_P41_03). All isolates from this cluster grew in M1 supplemented with GalNAc regardless of the presence or absence of pELF_USZ (Fig. S8B). We therefore hypothesized that their genome included another genetic determinant conferring this trait. A query for homologous gene clusters revealed a cluster including both the uptake system (all four PTS components) and a partial degradation pathway on their chromosome (Fig. S8C and Data Set S1, Tab 8). We presumed this cluster, absent from the genome of *cluster 10* isolates, to underlie *cluster 6* isolates’ GalNAc utilization.

To verify whether pELF_USZ itself was sufficient for GalNAc utilization and, in parallel, investigate its transferability, we performed a filter-mating experiment, using VRE53_P46 as donor and BM4105RF as recipient. The latter did not carry any plasmid and could not utilize GalNAc for growth. Upon antibiotic selection and accumulation in M1 supplemented with GalNAc, a descendant that could utilize GalNAc for growth was obtained (Fig. 5C). Whole-genome sequencing confirmed that the presence of pELF_USZ was the only difference between this transconjugant and the recipient, and they had comparable growth dynamics in M1 supplemented with glucose (Fig. 5C).

In rich liquid medium, the *outbreak clade* isolates had a significantly lower maximal growth rate than the *ancestral* isolates (Fig. S8D). The fact that the maximal growth rate of the only clade member without pELF_USZ (VRE67_P60) was in the same range as the others suggested that this difference was not associated with the plasmid. Consistent with this, the maximal growth rates of BM4105RF and BM4105RF+pELF_USZ did not differ significantly (Fig. S8D).

In conclusion, pELF_USZ, which was transferrable by conjugation, was sufficient, but not necessary, to confer the ability to utilize GalNAc.

## DISCUSSION

In this study, we characterized transmission networks of VREfm clones at the Swiss tertiary care hospital USZ between October 2014 and February 2018. An ST-203 VREfm clone was repeatedly isolated from several patients over a time span of 29 months. During this period, a few mutations accumulated in its core genome, including an SNP leading to decreased daptomycin susceptibility. Furthermore, the clone acquired a linear plasmid, named pELF_USZ, which conferred the ability to use GalNAc for growth.

Since genomic approaches have proven their superiority over traditional molecular typing methods for epidemiological surveillance of nosocomial pathogens (45, 46), their implementation in routine settings is in progress. Between October 2014 and February 2018, the standard approach for VREfm surveillance at USZ consisted of the combination of traditional molecular typing methods with epidemiological criteria. The phylogenomic analysis presented here confirmed close relatedness across all isolates presumed to be involved in transmission events by this standard surveillance approach. Furthermore, it revealed relatedness across isolates for which no epidemiological link had been established. It must be emphasized that genetic relatedness cannot establish the route, localization, and directness of transmission events.

We showed that some patients carrying the same clones/resistance determinants that were not epidemiologically linked at the USZ had been transferred from hospitals of the same country. We concluded that these cases likely corresponded to sporadic sampling of external transmission networks. This could be further supported in the case of *cluster 3*, which represented a *vanB*/ST-117 clone, reported to predominate over other clones in Germany in the last few years (29, 47–49).

The maximum of 12 SNPs differentiating the 31 isolates of the *persistent clone* sampled over 29 months represents a low diversity compared to that characterizing other clones of the collection. This indicates a recent emergence, consistent with the estimation by Bayesian phylogenetic analysis of a most recent common ancestor dating to 3 months before the first sampling event (credibility interval, 1 to 6 months). The substitution rate of this clone was estimated at 3.8 substitutions per genome per year, which is slightly lower but in the range of previously observed between-host evolutionary rates for E. faecium: five and seven substitutions per genome per year (15, 16), respectively.

The only epidemiological link between the 2018 outbreak and earlier sampling of the *persistent clone* was patient 14, from whom the *persistent clone* was sampled in 2016 (VRE14_P14_01) and again in 2018 (VRE69_P14_02). However, there was no evidence for prolonged carriage by this patient, who tested negative for VREfm carriage three times between the two sampling events. The clone might as well have persisted in unsampled individuals or in the environment. Sampling of potential environmental contamination and systematic screening for carriage would probably have elucidated further epidemiological links (18).

The main strength of this study was the functional genomic analysis of longitudinally sampled isolates, allowing the identification of natural changes adopted by an individual clone persisting in the hospital environment. Indeed, while long-term persistence of VREfm clones within health care systems has been previously described (15, 16), their microevolution, including horizontal gene transfer across them, has not been investigated in detail to date (21, 50).

This led to a first observation: a variant of the *persistent clone* with decreased daptomycin susceptibility spread among 17 patients during the outbreak of 2018. In the first place, this variant was likely selected within host, either during treatment of one of the sampled individuals or in the off-target carriage population of any unsampled individual, as suggested by Kinnear et al. (51). However, while within-host evolution of variants with decreased daptomycin susceptibility is commonly reported, their clonal spread is rarely observed (52–54). One clone with a baseline daptomycin MIC of 3 to 4 μg/mL spread clonally in hospitals of the New York metropolitan area and was associated with daptomycin nonsusceptibility (MIC, ≥8 μg/mL) (32, 55, 56). In the case presented here, further mutations leading to daptomycin nonsusceptibility have not been observed: the *persistent clone* has not been further identified by genomic surveillance up to the present.

During the same 13-month sampling gap, the *persistent clone* underwent the second genetic change highlighted in this study: the acquisition of a novel plasmid harboring carbohydrate utilization genes. Such genes have been repeatedly identified as determinants of adaptation to specific niches for enterococci, yet the specific function of most of the concerned operons is unknown. In the few cases in which this was specifically investigated, it could not always be unveiled, e.g., the substrate of the mannose-family PTS enriched in the clade A1, PTS^clin^, remains to be elucidated (27).

In this study, we demonstrated that acquisition of the novel plasmid pELF_USZ conferred the ability to utilize GalNAc. Based on functional annotation, it is highly plausible that GalNAc was the substrate of the mannose-family PTS of pELF_USZ and could be degraded by the enzymes on the operon accompanying it. Indeed, these genetic elements, including the transcriptional regulators, are consistent with the GalNAc utilization pathway proposed for streptococci and *Firmicutes* (57). However, some isolates with a similar but incomplete set of genes on the chromosome were also able to utilize GalNAc (isolates of *cluster 6* without pELF_USZ), suggesting that not all these specific elements are essential. In future studies, the generation of targeted mutants may allow us to clarify the role and regulation of specific genes underlying this trait in E. faecium.

GalNAc is one of the primary components of the intestinal mucins. While E. faecium cannot degrade mucins or plant polysaccharides, it competes with other members of the gut microbiota for monosaccharide components released upon degradation by the anaerobes (58). Thus, we speculate that an E. faecium clone able to utilize GalNAc could colonize the gut more efficiently under some conditions, i.e., specific compositions of gut bacterial community, affected by the patient’s underlying illness or antibiotic treatment. Such ecological relevance could be further explored by determining gut colonization dynamics of isogenic strains with and without pELF_USZ in an animal model.

We hypothesized that the uptake of the plasmid happened within the bacterial population of one host and conferred an advantage to the subpopulation concerned, resulting in fixation and subsequent spread to other hosts. The only *outbreak clade* isolate that did not harbor the plasmid, VRE67_P60, was derived from a wound infection of the only patient for whom no epidemiological link with the other patients of the outbreak could be established. This descendant might have lost pELF_USZ, or it might have stemmed from an ancestor dating prior to the plasmid acquisition.

The clone of *cluster 6* presumably took up pELF_USZ without gaining a growth advantage. In other words, the horizontal gene transfer across clones may have counteracted the effect of purifying selection. However, we did observe the loss of pELF_USZ during within-host persistence (patient 41; isolate 01 carried it and the two follow-up isolates did not), as theoretically expected in the absence of positive selection for the cargo genes (59, 60).

This plasmid exhibited a linear topology, in contrast to the traditional circular topology of bacterial plasmids. Linear forms have been found in both Gram-negative and Gram-positive species (61, 62), and while the latter mostly belong to the *Actinobacteria* phylum, two linear plasmids were recently identified in the genome of E. faecium clinical isolates in Japan (37, 38). The similarity of pELF_USZ and other uncircularized plasmid sequences with these linear plasmids suggests the existence of a conserved family of linear plasmids in enterococci. The faculty of this conserved plasmid backbone to harbor various ecologically relevant cargo genes in diverse clinical clones suggests that it plays a part in the continuous adaptation by horizontal gene transfer of this species. Further research is needed to assess epidemiological relevance as well as the replication and transfer mechanisms of these linear plasmids.

From the bacterial genomics perspective, eventual linear plasmids would have been difficult to detect until now, as the short-read sequencing technology does not allow us to reconstruct genome structure. This is now possible with the long-read sequencing technology. However, sequences that are uncircularized upon hybrid assembly tend to be considered sequencing or assembly artifacts and might be disregarded in downstream analyses. Our finding encourages the consideration of uncircularized sequences as plausible biological occurrences.

So far, genomic surveillance has focused on the signal provided by vertical evolution of pathogens to track spread of clones. Recent studies started using the high resolution offered by genomic surveillance to scrutinize dissemination dynamics of plasmids carrying resistance determinants within health care systems (50, 63, 64). Of note, relying on a single colony sampled from the VREfm colonizing population of a few patients at isolated time points limited the resolution at which horizontal gene transfer dynamics could be explored in our study.

To sum up, by combining epidemiological, functional genomics, and evolutionary perspectives, we observed evidence for clonal and horizontal spread of a plasmid providing an innovative nutrient utilization trait, which could be adaptive under specific conditions. We envision that further studies at the microevolutionary scale will allow us to understand adaptation strategies that contribute to the success of the hospital-adapted pathogen VREfm.

## MATERIALS AND METHODS

### Isolate collection and epidemiological characterization.

From October 2014 to February 2018, 69 VREfm isolates were sampled from 61 patients during routine diagnostic and surveillance screenings at the USZ, a tertiary care facility in Switzerland. Surveillance screenings were carried out for exposed patients. Patients were considered exposed if they shared the same room as an index patient for more than 24 h on a normal ward or were in a neighboring bed of an index patient for more than 24 h on the intensive care unit. In addition, upon identification of multiple cases in the same ward, all patients hospitalized in that ward were considered exposed.

All isolates were typed with MLST according to the PubMLST typing scheme and PFGE of SmaI digests according to standard protocols. Transmission events were suspected by routine surveillance if isolates recovered from patients who had been exposed to each other as defined above shared the same MLST and PFGE pattern. Moreover, isolates sampled longitudinally from an individual were also considered to be related if they showed the same MLST and PFGE pattern and no more than one intermediary negative screening. The necessity of a formal ethical evaluation was waived by the Zurich Cantonal Ethics Commission (req-2021-00037).

### DNA isolation, whole-genome sequencing, and assembly.

The DNeasy blood and tissue kit (Qiagen) was used for DNA extraction from single colonies inoculated in brain heart infusion broth (BHI; Bacto) and grown overnight (ON) at 37°C, with shaking at 220 rpm. DNA libraries were prepared with the NEBNext Ultra kit (New England BioLabs) and sequenced on a MiSeq instrument (Illumina) with 600-cycle paired-end runs. The quality of the paired-end reads was assessed using FastQC 0.11.15 (https://www.bioinformatics.babraham.ac.uk/projects/fastqc/), and low-quality reads were filtered out with Trimmomatic 0.36 (65). *De novo* assemblies were constructed with SPAdes 3.10 (66) and final assemblies evaluated with Quast 3.1 (67).

Four isolates were selected for long-read sequencing with Oxford Nanopore Technology (ONT). The Gentra Puregene kit (Qiagen) was used to extract high-molecular-weight DNA, and the four DNA samples were pooled onto one MinION flow-cell and sequenced on the GridION X5 system. Hybrid and long-read-only assemblies were generated with Unicycler 0.4.8 (36).

### Phylogenomic analyses.

All assemblies, including those of the 73 reference genomes (2) were annotated with Prokka 1.13 (68). Two pangenomes were constructed with Roary 3.12 (69): one including the 69 genomes of this study and all 73 reference genomes and another including the 69 genomes of this study and the 21 reference genomes belonging to clade A1. We passed the -e parameter to generate core gene alignments with MAFFT. These alignments, of 1,131,323 bp and 1,546,174 bp, respectively, were used to build maximum likelihood phylogenetic trees with FastTree 2.1.10 (70). Phylogenomic clusters were defined based on the 1,546,174-bp core gene alignment, using a maximum pairwise distance cutoff of 102 SNPs, so that, upon within-cluster full-genome comparison followed by recombination filtering, <100 SNPs differentiated pairs of isolates of the same cluster. To assess the sensitivity of our results on the chosen threshold, we summarized maximum pairwise distances and all pairwise distances in Data Set S1, Tab 3 and Tab 4, respectively, in the supplemental material.

Within-cluster comparisons were performed by mapping all short-read sequences from a cluster to one *de novo* assembly with Snippy 3.2 (https://github.com/tseemann/snippy). The alignment resulting from *snippy-core* (including both variant and invariant sites) was used as input to Gubbins 2.3.1 (71) to identify and filter SNPs having arisen from recent recombination events. Variant sites were extracted with SNP sites (72) from the global core gene alignment; the within-cluster alignment prior to recombination filtering and the recombination-filtered SNP alignment. Corresponding pairwise distance matrices were constructed with snp-dists (https://github.com/tseemann/snp-dists). Maximum likelihood phylogenetic trees were built with FastTree 2.1.10 (70) based on the recombination-free SNP alignments, and temporal signal was assessed with TempEst 1.5.1 (73).

To establish a catalogue of all mutations differentiating the 31 isolates of the *persistent clone* and identify their effect, variant-calling was performed with Snippy 3.2 using as reference the hybrid assembly of USZ_VRE5_P5 as well as the annotated reference genome Aus0004 (GenBank accession no. GCA_000250945.1) (74).

### Characterization of the vancomycin resistance transposon.

The SRST2 0.2.0 tool (75) was used to screen for resistance genes of the ARGannot_r3 database in each genome. Genomes including *vanA* genes were mapped to the transposon Tn*1546* of BM4147 (GenBank accession no. M97297.1) (76, 77) and those including the *vanB* genes to the transposon Tn*1549* of Aus0004 (nucleotide positions 2835430 to 2869240 of GenBank accession no. CP003351) (14) using Snippy 3.2 as well as bwa-mem (78) and the SAMtools suite (79). The alignments (coverage and SNPs) were inspected with IGV (80). To assess the incorporation of insertion sequences and determine their position, contigs of the short-read assemblies that mapped to these references were queried against the Insertion Sequence database (https://github.com/thanhleviet/ISfinder-sequences, 2020-Oct version, compiled from ISFinder [81]).

### Bayesian phylogenetic inference.

To estimate the height of the phylogeny of the *persistent clone* (Fig. 2B) and the clock rate, Bayesian Markov chain Monte Carlo (MCMC) phylogenetic inference implemented in BEAST 2.6.3 (82) was performed. The 32-bp recombination-free SNP alignment corrected for compositional bias was used as input. The analysis employed a Hasegawa–Kishino–Yano (HKY) substitution model, a strict clock model, and a birth-death skyline serial model (BDSKY) for the tree prior (83). The sampling proportion prior was centered at 1%. The output and chain convergence were examined with Tracer 1.6.0 (84), and a maximum clade credibility (MCC) tree was built with the TreeAnnotator utility of BEAST. The robustness of the posterior densities when changing the sampling proportion prior to center around 10% was confirmed.

### MIC test.

Daptomycin and fusidic acid susceptibility were determined using test strips (Text S1).

10.1128/mbio.03771-21.2 TEXT S1 Detailed laboratory protocols for minimum inhibitory concentration test, growth assessments and filter-mating experiments. Download Text S1, DOCX file, 0.03 MB. Copyright © 2022 Boumasmoud et al. 2022 Boumasmoud et al. https://creativecommons.org/licenses/by/4.0/ This content is distributed under the terms of the Creative Commons Attribution 4.0 International license.

### Plasmid characterization.

To investigate the topology of pELF_USZ, PFGE of total DNA of four representative isolates, either undigested or digested 15 min with S1 nuclease (Promega), was carried out as described by Freitas et al. (85). Contig coverage and dead ends of both the short-read and hybrid assembly graphs were inspected using Bandage 0.8.1 (86). Hybrid assembly was compared with long-read-only assembly and alignments visualized using Easyfig 2.2.5 (87). DNA folding of the 100-bp stretch at the center of the inverse repeats of the left extremity of pELF_USZ was inspected with mfold 3.6 (88). pELF_USZ and the other linear plasmids were screened for prophage sequences, replicons, and relaxases with the web server tools PHASTER (89), PlasmidFinder 2.1 (90), and MOBscan (91). Putative functions of CDSs with a putative product were determined by querying against the nonredundant protein sequences (nr) database and the eggNog 5.00 (92).

For the comparison of plasmid sequences in the two complete assemblies (USZ_VRE32_P32 and USZ_VRE53_P46), two similarity indexes were considered: the percent average nucleotide identity (ANI), which is based on alignment and thus only considers the core (93), and the mash distance, which relies on k-mers comparisons and thus also reflects differences in sequence length (94).

To identify gene clusters homologous to the carbohydrate utilization system of pELF_USZ, the *search* function of cblaster (95) was used to query amino acid sequences of the 11 CDSs (*yvoA*, *agaS*, *lacD2*, *lacC*, *frlR*, *bga*, *levE*, *sorC*, *manZ*, *manX*, and *immR*) against short-read assemblies of *cluster 10* and *cluster 6* isolates. To identify the genomic location of the hits, the query was also performed against the complete assembly of VRE32_P32. The *extract_clusters* function then was used to obtain .gbk files of each cluster, and their comparison was plotted with clinker (96).

### Growth curves.

For the growth screening assay, Phenotype MicroArrays PM1 and PM2A (Biolog Inc.) were used. Since no growth was obtained in the defined medium proposed by the manufacturer (inoculating fluid IF0a GN/GP plus additive solution), the medium M1 (10 g tryptone and 0.5 g yeast extract into 1 liter phosphate-buffered saline), designed for minimal growth of E. faecium by Zhang et al. (44), was employed (Text S1). To validate the role of pELF_USZ in GalNAc utilization, the 31 isolates of the *persistent clone*, BM4105RF and BM4105RF+pELF_USZ, were cultivated in M1 and M1 supplemented with 0.1% GalNAc (Sigma-Aldrich). Moreover, to assess their maximal growth rate in nutrient-rich liquid medium, they were grown in BHI broth (Text S1).

### Filter-mating experiment.

The isolate VRE53_P46 was used as the donor and BM4105RF, a plasmid-free rifampicin- and fusidic acid-resistant strain, as recipient (97). The mating protocol proposed by Werner et al. (98) was followed (Text S1). The presence of pELF_USZ in the genome of the isolated transconjugant was confirmed by PCR, amplifying a fragment of the *agaS* gene with the primers agaS_f 5′-CCATCATTTATCAATCACCTGTGCA-3′ and agaS_r 5′-GTGTCTAAAGCCCATCGATGAATCA-3′. Moreover, the recipient and the transconjugant were subjected to whole-genome sequencing and comparative genomics, as described above.

### Data availability.

Illumina paired-end reads of the 69 VREfm isolates; Nanopore reads and hybrid assemblies of VRE5_P5, VRE53_P46, VRE67_P60, and VRE32_P32; and Illumina paired-end reads of BM4105RF and BM4105RF+pELF_USZ are available through European Nucleotide Archive project no. PRJEB44616. pELF_USZ was assigned the GenBank accession no. OU016038.1. Raw data for growth curves and corresponding figures can be found at https://www.doi.org/10.6084/m9.figshare.15406335.
