# Supplementary figures and images for: Genomic Surveillance of Vancomycin-Resistant Enterococcus faecium Reveals Spread of a Linear Plasmid Conferring a Nutrient Utilization Advantage
Source: mBio. 2022 Mar 28;13(2):e03771-21. doi: 10.1128/mbio.03771-21 (PMC9040824; doi:10.1128/mbio.03771-21)

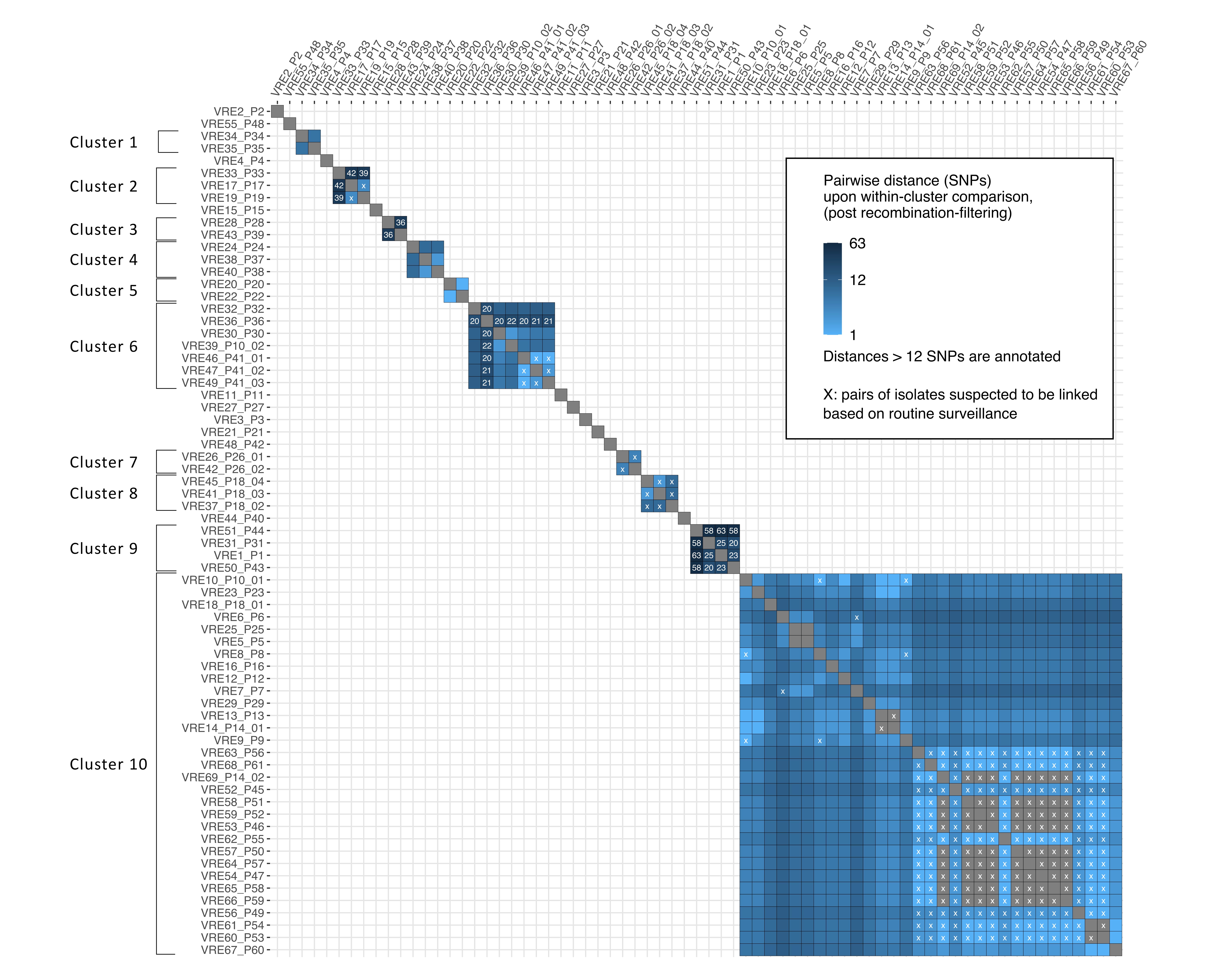

Supplement: FIG S2 [file mbio.03771-21-sf002.jpg]

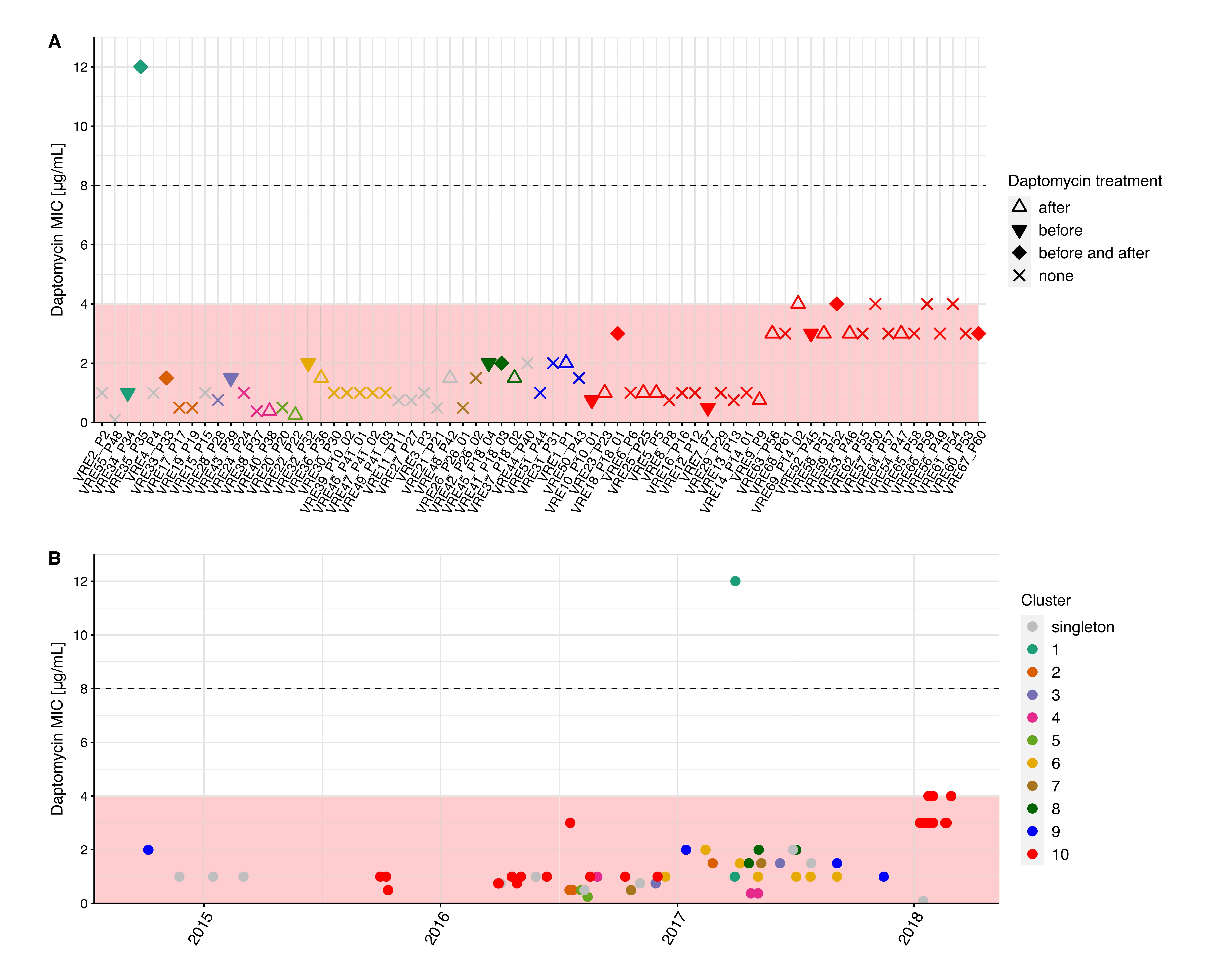

Supplement: FIG S3 [file mbio.03771-21-sf003.jpg]

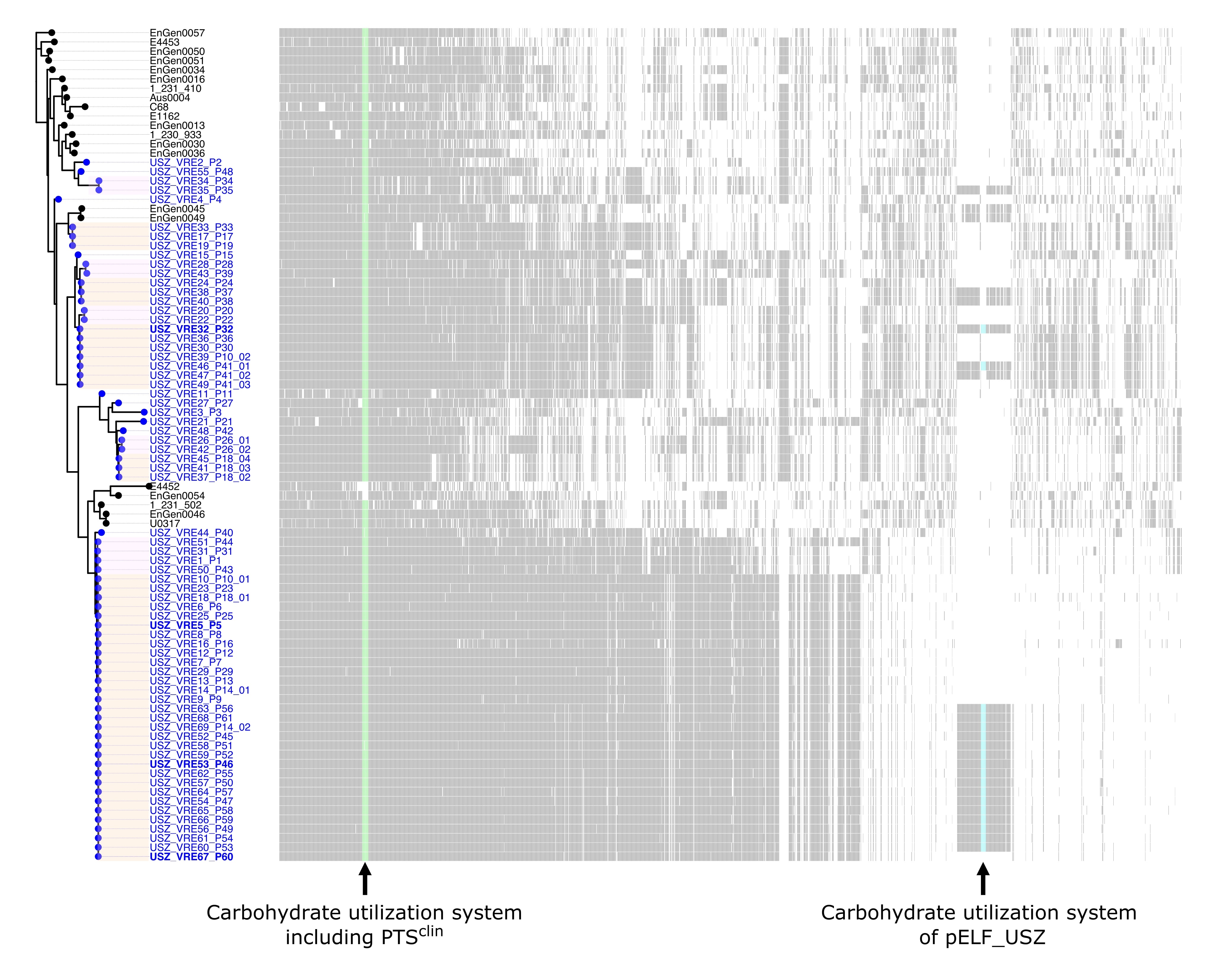

Supplement: FIG S4 [file mbio.03771-21-sf004.jpg]
