# Supplementary material for: Genomic Surveillance of Vancomycin-Resistant Enterococcus faecium Reveals Spread of a Linear Plasmid Conferring a Nutrient Utilization Advantage
Source: mBio. 2022 Mar 28;13(2):e03771-21. doi: 10.1128/mbio.03771-21 (PMC9040824; doi:10.1128/mbio.03771-21)
Supplement: TEXT S1 [file mbio.03771-21-s0001.docx]

**Supplemental Methods for**

**Genomic surveillance of vancomycin-resistant *Enterococcus faecium* reveals spread of a linear plasmid conferring a nutrient utilization advantage**

Mathilde Boumasmoud^1^, Vanina Dengler Haunreiter^1^, Tiziano A. Schweizer^1^, Lilly Meyer^1^, Bhavya Chakrakodi^1^, Peter W. Schreiber^1^, Kati Seidl^1,2^, Denise Kühnert^1,3^, Roger D. Kouyos^1^ and Annelies S. Zinkernagel^1, *^

1. Department of Infectious Diseases and Hospital Epidemiology, University Hospital Zurich, University of Zurich, 8091, Zurich, Switzerland
2. Present address: Department of Pathology and Molecular Pathology, University Hospital Zurich, 8091, Zurich, Switzerland
3. Present address: Max Planck Institute for the Science of Human History, 07745, Jena, Germany

##

## Minimum inhibitory concentration test

Daptomycin and fusidic acid MICs were determined using test strips (Liofilchem). The first contained Ca2^+^ in addition to the daptomycin concentration gradient. The strips were placed on Mueller-Hinton II agar plates that had been inoculated with a cotton swab dipped into a 0.5 McFarland suspension. The inhibition zone was inspected after 24 h incubation at 37 °C.

## Growth assays

For the growth screening experiment, bacteria were inoculated directly from Columbia Sheep Blood agar plates (CSB, BioMérieux) into PBS at an optical density (OD) of 0.1. A mix containing 880 μl of this suspension, 11 mL of M1 and 120 μl Dye Mix H (BioLog Inc) was prepared, and 100 μl were transferred in each of the 96 wells of the PM microplates. The absorbance at 600 nm (OD_600_) was monitored every 10 min for 48 h with the plate‑reader instrument Tecan infinite 200, while incubating at 37 °C under constant shaking (orbital, amplitude 3 mm and 5 s before measuring: linear, amplitude 1.5 mm).

Growth curves were calibrated by subtracting the baseline, i.e., the first OD_600_ value measured for each well. For each isolate and condition, the area under the average curve from three replicates was calculated. The area under the curve (AUC) for the blank, i.e., M1 only, was 133.8 ± 6.5. Conditions in which the AUC was ≥ 230 + the within-biological-replicates standard error were considered as growth permissive.

For growth assays in BHI or M1 medium supplemented with N-Acety-D-Galactosamine (GalNAc), bacteria were inoculated in the respective media to achieve a final OD of 0.01. Technical triplicates of 200 μl from this suspension were transferred in 96 flat-wells microplates for cell culture (TPP). The OD_600_ was monitored every 10 min for 24 h, with conditions, instrument and settings described above. To determine maximal growth rate, growth rates were calculated for each 1 h interval, using $\frac{\ln\left( A_{f} \right)-\ln\left( A_{i} \right)}{(t_{f}-t_{i})\text{ }}$, where t and A represent the timepoint and absorbance, and the indexes *i* and *f* stand for initial and final.

## Filter-mating experiment

The donor and recipient were grown in BHI broth ON at 37 °C, shaking at 220 r.p.m. They were then diluted 1:20 into fresh BHI medium and allowed to regrow to exponential phase, at 37 °C, shaking at 220 r.p.m, till reaching ~10^8^ CFU/mL. The two cultures were then pooled 1:1 (2 x 1mL) and centrifuged at 14,000 g for 2 min. The pellet was washed twice and finally resuspended in 1mL PBS. Of this, 100 μl were plated onto a 0.2 μm polycarbonate membrane filter (Isopore) placed on BHI agar, air-dried and incubated ON at 37 °C. The mix of donor, recipient and presumably transconjugants was then harvested from the membrane, placed into 20mL PBS and vortexed. A mix of recipient and presumably transconjugants was selected by plating on BHI agar containing 30 μg/mL rifampicin and 20 μg/mL fusidic acid. Cultivation of the presumed mixed population in M1 supplemented with 0.1% GalNAc resulted in significant growth, indicating that a subpopulation of transconjugants was indeed part of the inoculum, since the recipient could not grow on GalNAc. A single colony was subsequently isolated from the transconjugants-enriched population.
